# Optimization of the Inverted Emulsion Method for High‐Yield Production of Biomimetic Giant Unilamellar Vesicles

**DOI:** 10.1002/cbic.201900529

**Published:** 2019-10-11

**Authors:** Akanksha Moga, Naresh Yandrapalli, Rumiana Dimova, Tom Robinson

**Affiliations:** ^1^ Theory & Bio-Systems Department Max Planck Institute of Colloids and Interfaces Potsdam-Golm Science Park 14424 Potsdam Germany

**Keywords:** biomimetic, bottom-up synthetic biology, giant unilamellar vesicles, inverted emulsion, phase transfer, physiological buffers

## Abstract

In the field of bottom‐up synthetic biology, lipid vesicles provide an important role in the construction of artificial cells. Giant unilamellar vesicles (GUVs), due to their membrane's similarity to natural biomembranes, have been widely used as cellular mimics. So far, several methods exist for the production of GUVs with the possibility to encapsulate biological macromolecules. The inverted emulsion‐based method is one such technique, which has great potential for rapid production of GUVs with high encapsulation efficiencies for large biomolecules. However, the lack of understanding of various parameters that affect production yields has resulted in sparse adaptation within the membrane and bottom‐up synthetic biology research communities. Here, we optimize various parameters of the inverted emulsion‐based method to maximize the production of GUVs. We demonstrate that the density difference between the emulsion droplets, oil phase, and the outer aqueous phase plays a crucial role in vesicle formation. We also investigated the impact that centrifugation speed/time, lipid concentration, pH, temperature, and emulsion droplet volume has on vesicle yield and size. Compared to conventional electroformation, our preparation method was not found to significantly alter the membrane mechanical properties. Finally, we optimize the parameters to minimize the time from workbench to microscope and in this way open up the possibility of time‐sensitive experiments. In conclusion, our findings will promote the usage of the inverted emulsion method for basic membrane biophysics studies as well as the development of GUVs for use as future artificial cells.

## Graphical abstract

**The inverted emulsion method** can produce giant unilamellar vesicles in physiological buffers with encapsulation of large (bio)molecules. Our study focuses on the optimization of the parameters for maximizing yields including; lipid concentration, centrifugal force, incubation time, aqueous emulsion volume, and buffers. We also investigate the biomimetic properties and functionality of the membranes to facilitate the wider use of the method.

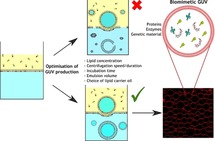

## Introduction

Minimal cell research has gained considerable interest in recent years.^1^ Successful realization of which could open up the possibilities of not only a deeper understanding of biological cell complexities but also for engineering new artificial cells designed with specific tasks in mind. The “top‐down” approach aims to reach this goal through the modification of pre‐existing organisms. The “bottom‐up” approach, on the other hand, hopes to achieve this goal by assembling an artificial cell from individual non‐living components.^1^ The latter method, although it may take longer to reach the goal, gives us the opportunity for complete control of the system, which may lead to a broader range of future applications.

Theoretically, the construction of a minimal cell from the bottom‐up should be possible by mimicking and redesigning what we see in nature using individual components, such as sugars, lipids, proteins, and genetic material.^2^ Before these steps can take place, a suitable compartment system to contain these materials should be established. Among all the choices, lipid vesicles remain the most likely candidates to succeed. These vesicular structures are composed of phospholipids and come in various sizes, such as small unilamellar vesicles (SUVs) below 100 nm diameter, large unilamellar vesicles (LUVs) from 100—1000 μm, and giant unilamellar vesicles (GUVs) between 1 and approximately 100 μm.^3^, ^4^, ^5^ GUVs are considered the gold standard of cellular mimics owing to their similarity in size to that of a mammalian cell. Moreover, with advances in technologies, such as microfluidics for their production^6^ and handling,^7^ GUVs are looking like an increasingly more attractive option.

The challenge lies in not only encapsulating large biomolecules inside giant vesicles (something which not all GUV preparation techniques can achieve), but also in establishing a reliable method for their production. Having biomolecules, such as actin networks or proteins in general on the inside of lipid vesicles is an essential requirement for the bottom‐up construction of artificial cells. High protein encapsulation is also of interest for biophysical studies when their interaction with inner leaflet plasma membranes lipids is under investigation.^8^, ^9^, ^10^, ^11^ Moreover, techniques, which allow encapsulation of low samples volumes are often desirable. Unfortunately, conventional techniques do not readily yield the encapsulation of large biomolecules and do not offer control over size or yield.^12^, ^13^, ^14^ Recently, microfluidic systems have been demonstrated as excellent platforms for the preparation of monodisperse liposomes with high precision lossless encapsulation of biomolecules.^15^, ^16^, ^17^ The most common methods being double emulsion templating by co‐axially aligned glass capillaries.^18^, ^19^, ^20^ Alternative microfluidic techniques include cDICE,^21^ jetting,^22^ and pico‐injection.^23^ For a more detailed review of these methods please refer to references 17 and 24. Although microfluidic devices do yield GUVs, which are more monodisperse in size and with higher encapsulation efficiencies, their use involves more complex instrumental or time‐consuming setups, which are not always available to researchers.^6^, ^25^, ^26^, ^27^ On the contrary, the bulk inverted emulsion‐based method has shown great promise in encapsulating macromolecules, such as polymers,^28^ DNA,^29^ enzymes,^30^ cells,^31^ and even micron‐sized particles.^32^ The method can also be used to produce GUVs with complex multicomponent lipid mixtures allowing them to be used as biomimetic membrane models.^33^, ^34^

Despite this, the method suffers from a few drawbacks resulting in poor yields or insufficiently large GUVs. For this reason, very few groups have adopted the method and often prefer more established techniques, such as electroformation or gentle hydration,^4^, ^5^, ^14^ even if these choices limit the range of experimental possibilities. This paper aims to address this issue by optimizing each of the required steps in the inverted emulsion method to maximize the yield and reliability. Normally, it is performed in Eppendorf tubes resulting in one preparation of GUVs. Not only is this low‐throughput, but in the absence of proper optimization, results in very low vesicle yields. Recently, we presented work in which we demonstrated that microtiter plates are ideal for performing this method as it allows multiple parallel experiments.^29^ We take advantage of this setup within this work and perform repeatable parallel experiments to allow optimization.

**Figure 1:**
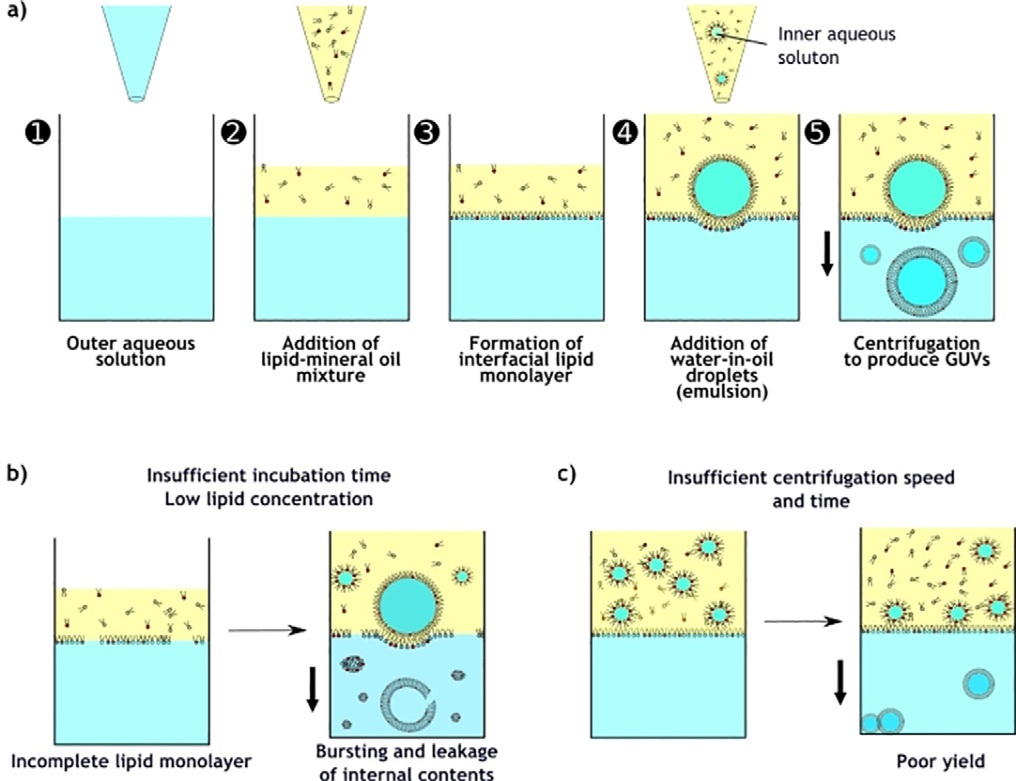
Overview and the pitfalls of the inverted emulsion method. a) Schematic representation of the five steps of the inverted emulsion method with the ideal GUV production. Parameters, such as b) low lipid concentrations and insufficient incubation times may result in an incomplete interfacial lipid monolayer that can compromise overall GUV yield and size. c) Centrifugation speed and duration also have an impact on the morphology and yield of the GUVs as the droplets may remain in the oil phase or at the interface.

The typical procedure (Figure 1 a) starts with the initial dissolution of a specific concentration of a chosen lipid mixture in oil (such as mineral oil or mixture of multiple oils) that is later added on top of an aqueous solution to form an oil–water interface. The lower aqueous solution will eventually become the outer environment of the GUVs (typically a glucose solution). At the same stage, a specific volume of denser aqueous solution is added to the lipid–oil phase (in a separate vial) to produce a water‐in‐oil emulsion. This aqueous phase will become the inner compartment of the GUV and will therefore contain the solutes to be encapsulated. Owing to their amphipathic nature, lipids present in the oil phase self‐assemble at the interfaces (both the emulsion and the lower interfacial layer, see Figure 1 a) to form lipid monolayers. The emulsion is then added on top of the interface and a centrifugal force is applied to assist the movement of denser water‐in‐oil emulsion droplets through the interfacial lipid monolayer. This process will result in the simultaneous wrapping of the second monolayer of lipids around the droplets and the formation of GUVs in the lower aqueous phase. Serious consideration should be made to ensure the fact that the oil used to solubilize the lipids can remain in between the lipid leaflets, which can alter the natural physical properties of the membrane.^35^, ^36^, ^37^ Any protocol, which uses an oil phase to form lipid membranes should have the membrane inspected. This could be one of the major reasons for lack of widespread acceptance and usage of this method, even though a couple of studies have shown no significant changes in the membrane mechanics of GUVs prepared from inverted emulsions.^38^, ^39^ In addition to this, the method must be optimized for compatibility with a range of salt concentrations and pH values, as well as having charged/uncharged macromolecules inside or outside the GUVs to make them as biomimetic as possible.

To the best of our knowledge, an extensive systematic study of the various parameters that might affect the production of GUVs using this method has not been attempted previously. In this paper, we analyze the resulting vesicles with respect to their size and yield by thoroughly optimizing each preparation step. We investigate the significance of the applied force, centrifugation duration, the inner solution density/composition, the monolayer formation time, oil phase, lipid concentration, pH, droplet volume, and temperature. Importantly, we confirm the applicability of the GUVs by characterizing their membrane functionality and property by performing a membrane protein‐based permeation assay as well as bending rigidity measurements. Finally, the method can be performed within microtiter well plates for high‐throughput production and analysis.

## Results and Discussion

The multiple steps involved in this inverted emulsion method can lead to unseen errors (Figure 1 b and c) which may lead to low yields. So here, we optimize each step systematically to identify the most crucial ones and the effects they have on the morphology as well as the yield of the GUVs. Performing the method within microtiter plates allows us to run multiple parallel conditions for fast optimization. Here, we focus on the effects of density gradients of the aqueous solutions, the time and speed of centrifugation, the concentration of the lipids, the incubation time for the formation of lipid monolayer at the interface, the type of the inner solution, and the role of pH and temperature during the formation of the GUVs.

### Sugar‐based density gradients

Sugars, such as glucose and sucrose, have been extensively used in the preparation of giant vesicles to maintain isoosmotic conditions across the membrane and also for their relative inertness towards the lipid bilayer. In the case of the inverted emulsion method, the inclusion of a density gradient between the lipid oil and the inner aqueous solution is essential for the formation of GUVs. The water‐in‐oil droplet has to be transferred from the oil phase to the aqueous phase. Most natural oils are of low density compared to pure water and mineral oil, used primarily in this study, is no exception. Furthermore, there is another density gradient to be taken into consideration—the density difference between the inner aqueous solution and the outer aqueous solution. This allows the GUVs to settle at the bottom of the well not only for instant visualization but to avoid aggregation at the interface—made possible if the outer aqueous solution is less dense compared to the inner aqueous solution present inside the droplets (and eventually inside the GUVs). Some researchers have used gravity to provide the force to drive the droplets through the interface.^40^ This method of production not only requires a significant density gradient, but also needs a longer time for all the droplets to cross through the lipid monolayer to obtain a good yield. Alternatively, this process can be expedited by applying a centrifugal force, as performed herein.

**Figure 2:**
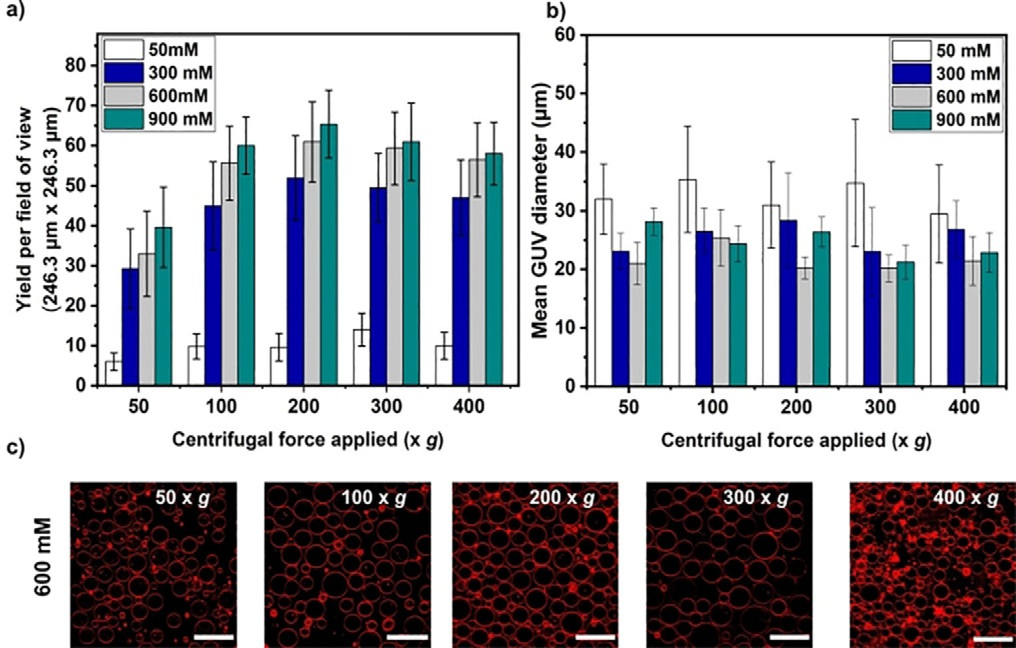
Yield and size distribution of 1‐palmitoyl‐2‐oleoyl‐*sn*‐glycero‐3‐phosphocholine (POPC) GUVs produced by varying the inner solution density and at multiple centrifugation speeds. a) Yields obtained for each concentration of inner sucrose solution tested at various centrifugation speeds. See the Experimental Section for yield calculation details. b) Size of the GUVs at different sugar concentrations and applied centrifugation speeds (*n*=3). c) Confocal images of GUVs containing 600 mm sucrose solution at various centrifugation speeds. Note that at 400 *g* bursting occurs. Here, the inner volume was 5 μL and the incubation time for the interface formation was 30 min with a lipid concentration of 400 μm. Scale bars: 50 μm.

From Figure 2 a, it is evident that there is a significant increase (at least ∼fivefold) in the number of GUVs produced when the inner sucrose solution concentrations are equal to 300 mm or above compared to 50 mm at any given applied centrifugal force. Note that glucose at an isoosmolar concentration is used as the outer solution throughout unless otherwise stated. Furthermore, increasing the centrifugal force applied for a specific time (here it was fixed at 3 min) is also effective in improving the number of GUVs produced until it plateaus at 400 *g*. However, it should be noted that high centrifugation speeds (above 300 *g*) result in clustering of GUVs at the cover glass and formation of lipid clumps due to bursting (Figure 2 c and Figure S1 in the Supporting Information). The average sizes of the giant vesicles produced tend to remain approximately the same for all the sugar concentrations and applied centrifugal forces tested. The range was between 20–35 μm in diameter, except for a 50 mm sugar concentration where some were less than 20 μm (Figure 2 b). Note that vesicles smaller than 10 μm are excluded from our analysis. We conclude that a denser inner solution promotes better yields due to a larger force experienced by each droplet. We speculate that the size variation for low sugars may be because larger GUVs do not survive or that the droplets did not make it through the interface. For the next experiments, we chose 600 mm sucrose as the optimal sugar concentration, that is, high yield, large GUVs, and minimum bursting. Although this ideal sugar concentration is higher than physiological osmolarities (∼260 to >400 msOm depending on the cell), more relevant concentrations of 300 mm also produce good yields.

### Centrifugal duration

**Figure 3:**
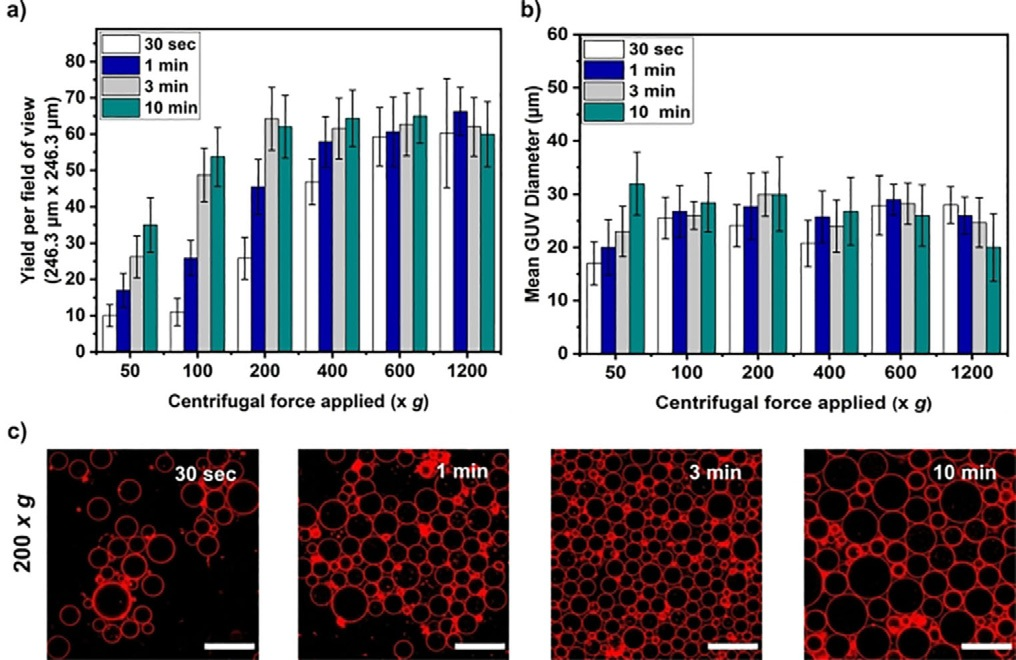
Plots of a) GUV yields and b) sizes produced at different centrifugation time periods and speeds. c) Confocal images of GUVs produced at 200 *g* centrifugation speed for various time periods. DiIIC18 was used as a membrane stain to visualize the GUVs. Note that error bars are taken from the standard deviations throughout (*n*=3). These results were obtained for fixed 600 mm sugar solutions, 5 μL inner volume, incubation time for the interface formation of 30 min, and with a lipid concentration of 400 μm. Scale bars: 50 μm.

From the above data it is evident that the inverted emulsion method is highly dependent on the density of the solutions used. Whereas a higher centrifugal speed for a short span of time might be enough to force all the droplets to pass through the lipid interface, our data suggest that it will also result in bursting and aggregation of the GUVs along the walls of the microtiter plate (see 1200 *g* centrifugal speed for 10 min in Figure S2). Therefore, we aimed at achieving good yields at lower speeds but with longer time durations as shown in Figure 3.

As expected, the yield increased with increasing duration of centrifugal force applied (Figure 3 a). This is to be expected as a longer applied force will increase the chances of the droplets crossing over the interface. Figure 3 a also shows that the yield of GUVs remains highest with a minimum of 3 min and above 200 *g*. This can be advantageous if speed of encapsulation is crucial. Furthermore, as the yield of the GUVs remained the same for higher forces it suggests that most of the droplets have passed through the interface (for this specific volume of emulsion droplets and solution density gradient). However, the plateau in yield could also be a result of 1) bursting of large vesicles at the glass well bottom due to higher forces and/or 2) the interfacial monolayer not having enough time to re‐seal before the next droplet arrives. We note that although the yield and size of the GUVs are superior from 600 *g* and above for all durations (see Figure S2 for images), fewer lipid aggregations occur at 200 *g* for 3 min (Figure 3 c). Considering this observation, we have chosen to use 3 min of 200 *g* centrifugation speed for the proceeding experiments.

### Volume of inner solution

**Figure 4:**
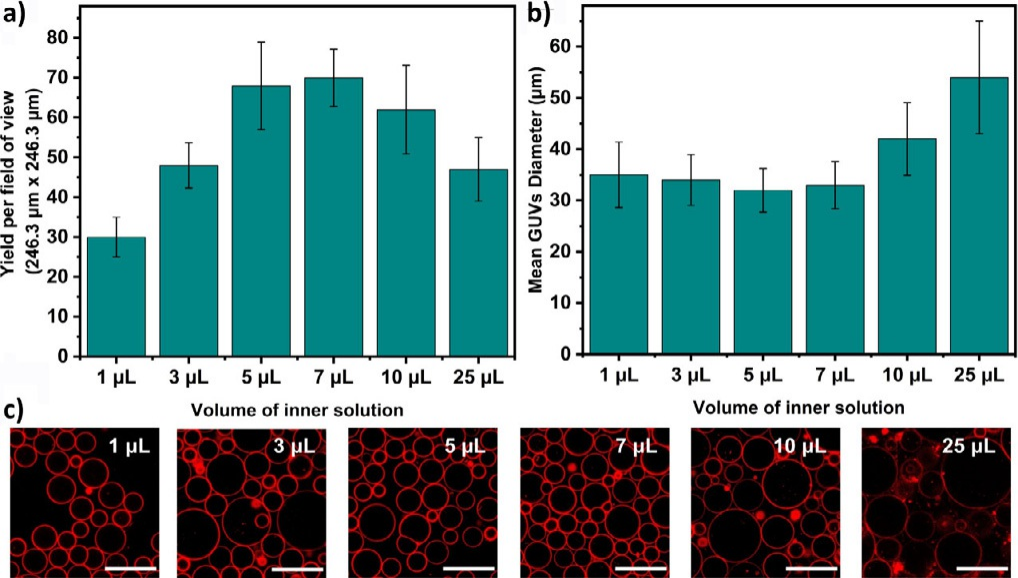
Plots presenting the effect that the inner solution volume used to make water‐in‐oil droplet emulsions has on a) the yield and b) the average size distribution of the GUVs. c) Confocal images of GUVs with 600 mm sucrose produced at 200 *g* speed for 3 min (*n*=3). Scale bars: 50 μm.

It is easy to predict that the GUV yield depends on the number of water‐in‐oil droplets. Therefore, we varied the volume of the inner aqueous solution used to produce the initial emulsion. Note that all other volumes were kept constant and the same total volume of 50 μL of emulsion was added. We used inner solution volumes from 1 to 25 μL and as Figure 4 shows, the yield increased with volumes between 5 and 10 μL. The size of the vesicles, however, peaked at 25 μL inner solution volume. It is reasonable to assume that the yield decreases with smaller volumes of the inner solution supplied for creating the emulsion, but it was unexpected that it decreases again at greater volumes. This phenomenon can be attributed to the fact that a higher volume of inner solution produces more droplets with increasing proximities to each other. This could result in a higher probability of spontaneous droplet fusion leading to larger vesicles but lower overall numbers. Indeed, this was observed in the case of 25 μL whose average diameter is 1.5‐fold more than the other conditions (Figure 4 b and c).

Consequentially, these results suggest an opportunity to tune the size of the GUVs to an extent by changing the volume of the inner solution to make the emulsion. Nevertheless, for the next experiments, we selected 7 μL for the inner solution volume as it produces the highest number of GUVs with the least variation in GUV size.

### Lipid concentration and incubation time

**Figure 5:**
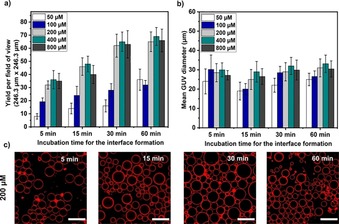
Plots of the a) yield and b) size distribution of the GUVs produced with various lipid concentrations in mineral oil and at different incubation time periods. c) Confocal images of GUVs with 600 mm sucrose solution produced at 200 *g* for 3 min with 200 μm lipid concentration in mineral oil (*n*= 3). Scale bars: 50 μm.

The concentration of solubilized lipids in the oil phase directly affects the time it takes to form the monolayers at the oil–water interfaces. This, in turn, puts a limit on the speed of the overall preparation time, which can be a disadvantage without optimization. Moreover, an incomplete lipid monolayer at the interface can directly affect the yield of the GUVs produced. For example, the aqueous interior of the droplets can make direct contact with the aqueous outer solution instead of the lipid monolayer at the interface resulting in the unwanted release of internal contents and reduction in the overall yield. Therefore, it is important to have a sufficiently assembled interfacial lipid monolayer before addition of the emulsion. In Figure 5, we explored the minimal required total concentration of lipids in the oil phase to help form a complete interfacial monolayer at the shortest possible time while still providing good GUV yields. For the various lipid concentrations tested (50, 100, 200, 400, and 800 μm), the GUV yield increases with the lipid concentration with any given period of incubation (Figure 5 a). Confocal images for 200 μm in Figure 5 c also show that a longer period of incubation results in better monolayer formation and yield (for images of other conditions see Figure S3). However, the data also reveals that if the lipid concentration is too low (<200 μm), it is not possible to increase the overall yield with a longer incubation time (Figure 5 a). Presumably due to the unavailability of lipids needed to cover the interfacial area of the well completely and to replenish it after droplet crossing. To confirm this assumption with a high degree of certainty, one would have to perform complementary simulations to investigate the minimum time required for the lipids to re‐assemble at the interface after droplet crossing. For higher lipid concentrations (200, 400, and 800 μm), the yield is similar and reached a plateau with a minimum of 30 min incubation time.

Considering this finding, for the next optimization we have fixed the interfacial lipid monolayer incubation time at 30 min and the lipid concentration at 200 μm. In addition, the range of sizes is similar to that of the experiments performed earlier in this work (∼20–30 μm in diameter), which demonstrates the robustness of the method in general (Figure 5 b).

### Effects of pH and temperature

**Figure 6:**
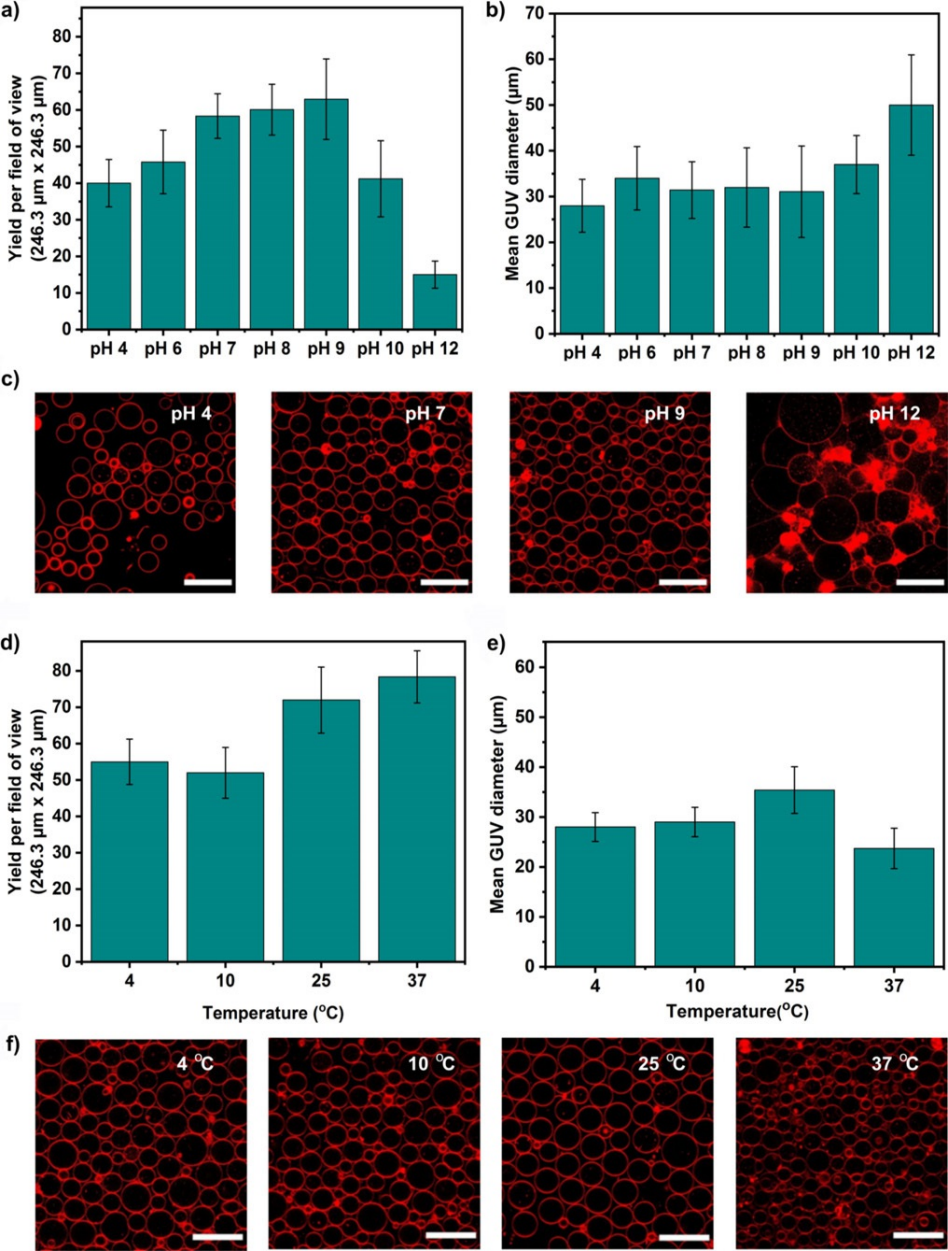
The yields and sizes of the GUVs produced at different a), b) pH and d), e) temperature conditions. Confocal images of these GUVs acquired for c) pH 4, 7, 9 and 12 and f) temperatures 4, 10, 25, and 37 °C (*n*=3). Scale bars: 50 μm.

pH and temperature play a crucial role in the functionality of many biological processes.^41^ As model membranes for biological cells, giant vesicles have been shown to be robust at various physiologically relevant pH and temperature ranges.^42^ Considering that this emulsion‐based method has the advantage of encapsulating large biomolecules such as enzymes,^30^ it is therefore possible to study the enzymatic activity of biologically relevant chemical reactions encapsulated within. To determine if it is possible to produce GUVs at such conditions, we prepared GUVs at various pH (inside and outside) and temperature conditions. Figure 6 shows the change in yield and size with varying temperatures and pH conditions. Yields moderately increased from pH 4 to 9 and decreased significantly at pH 12 (Figure 6 a). This can be explained due to the fact that at low and high pH values, the zwitterionic POPC lipid headgroup becomes more positively or negatively charged, respectively.^43^ It is known that charged lipids in monolayers repel each other, which may affect the formation and stability of the final GUVs. Moreover, at pH 12, the vesicles were larger with clustering and aggregation (Figure 6 b and c). This is most likely due to the hydrolyzing effect induced by hydroxide ions and possible degradation of the lipids at such high pH environments.^44^ In a further experiment to ascertain the greater applicability of the method for producing biomimetic compartments we used the buffering agents 4‐(2‐hydroxyethyl)‐1‐piperazineethanesulfonic acid (HEPES), Tris, and phosphate‐buffered saline (PBS) within inner solution only. Figure S4 shows that the method can produce GUVs containing these widely used buffers—something which is not always feasible with hydration‐based methods especially in the case of salt containing PBS solutions.

Unlike pH, temperature does not seem to have a drastic impact on the overall yield of the GUVs (Figure 6 d). The yield appears to increase marginally with temperature, at least for the one component POPC lipid composition implemented here. Although the maximum temperature used in this study (37 °C) yielded a higher number of GUVs, the average size of the GUVs ((23±4) μm) was smaller than the average size of the GUVs ((30±4) μm) produced at rest of the temperature conditions (Figure 6 e). Visually, the GUVs produced at all the temperatures tested were without defects or aggregations except at 37 °C where some small lipid clumps were seen (observed as bright red spots in Figure 6 f). From the results obtained, the inverted emulsion method can successfully produce biomimetic GUVs at different pH and temperature conditions—including physiological ones.

### Polymers as alternative density gradient agents

As already shown, the density of the inner solution is vital for producing GUVs using the inverted emulsion‐based method. A denser inner solution can easily pass through the oil phase and across the oil–water interface. Furthermore, a denser inner solution will help the GUVs settle down to the bottom of the well plate for better visualization and long‐term experiments. The above experiments encapsulated sugar‐based aqueous solutions to not only achieve this density difference, but also to osmotically balance the medium. The usage of sugar solutions might not be feasible if one has to encapsulate enzymes that can metabolize sugars or molecules that are sensitive to these carbohydrates. Moreover, encapsulating cells within GUVs for single‐cell analysis, which has gained some interest in recent years^31^ and high concentrations of sugars can affect cell viability or function.

**Figure 7:**
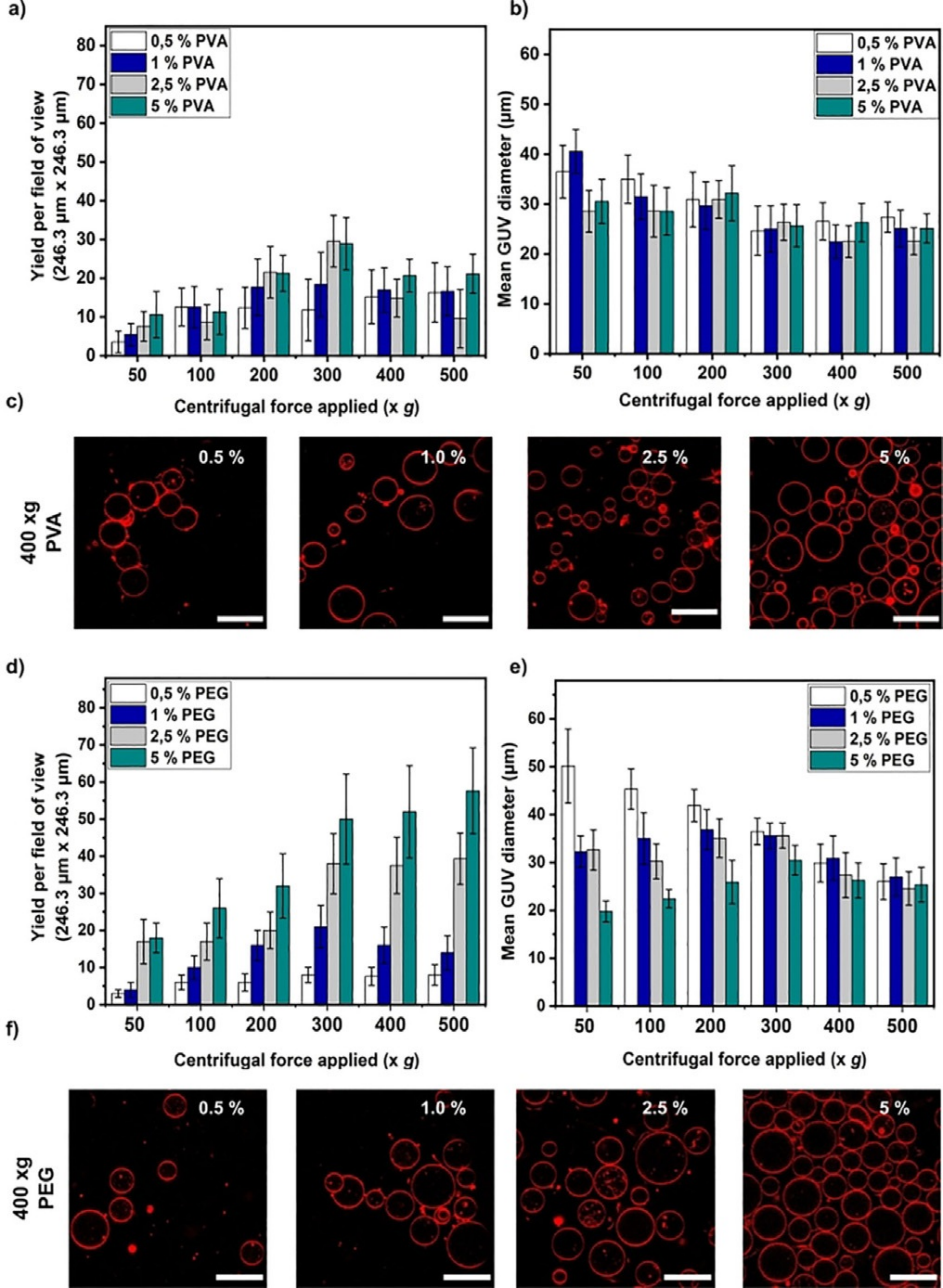
Plots of yield and size of GUVs obtained when different concentrations of polymers are encapsulated within the inner solution (with glucose outside). a), b) PVA and d), e) PEG. c), f) Representative confocal images of the GUVs produced for both the polymers PVA and PEG, respectively (*n*=3). Scale bars: 50 μm.

In such scenarios, alternative compounds that will not adversely affect the lipid bilayer have to be implemented. Considering this, we have turned to polymers, such as polyethylene glycol (PEG) and poly(vinyl alcohol) (PVA). PEG is a well‐known compound with high hydrophilicity and anti‐biofouling properties.^45^ Elsewhere, PVA is used as a coating material to grow GUVs using the gentle hydration method.^46^ Figure 7 depicts the yield and size of the GUVs produced at various polymer concentrations used as an inner solution (osmotically matched glucose outside) at different spin speeds. As expected, the GUVs yield increased with higher concentration of the polymers in the inner solution as the densities increased (Figure 7 a and d). Note that for concentrations of 0.5 to 5 % the osmolarities increased from 1 to 45 mOsm and 4 to 60 mOsm for PEG and PVA, respectively. At 400 *g*, the yield reached a plateau for both the polymers whereas the average size of the GUVs remained the same for all the concentrations at that spin speed. We also observed that the vesicles encapsulating PEG exhibit inward tubes, consistent with recent findings for stabilization of high spontaneous curvature by this polymer.^47^, ^48^ Surprisingly, from 300 to 500 *g*, the average size of the GUVs (∼25 μm) is approximately the same at all the polymer concentrations for both the polymers (Figure 7 b and e). When comparing data from both polymers, the overall yield is higher with PEG for all conditions tested (almost double for the highest polymer concentration at 5 *w*/*v* %). This could be due to density differences between the two polymers and/or that PVA can interfere with the lipid monolayer or bilayer by incorporating itself across the membrane. If the concentrations of 2.5–5 % PEG or PVA are not suitable for a particular application, lower concentrations also yield GUVs. From the confocal images, it is observable that the GUVs produced with PVA (Figures 7 c and S5) and PEG (Figures 7 f and S6) are morphologically similar to the GUVs produced by using sugar solutions (Figure 2 c) except that the overall yield is lower. Overall, the use of polymer solutions instead of sugar solutions to produce GUVs is possible using the inverted emulsion method and PEG is a more promising candidate.

### Membrane composition and functionality

We aimed to assess the membrane composition for any possible presence of oil in the lipid bilayer of the GUVs. This is important for the widespread acceptability and usage of this technique. Previously, researchers have performed alpha‐hemolysin‐based assays on GUVs produced by using microfluidic method to confirm oil‐free membranes, unilamellarity and thus, functionality.^49^ We have performed similar experiments with calcein dye‐filled GUVs produced using the inverted emulsion method. The plot presented in Figure S7 indicates that over a period of 30 min, there is a decrease in the calcein fluorescence intensity inside the GUVs in the presence of alpha‐hemolysin. This suggests that the lipid bilayer is oil‐free enough for the membrane protein to incorporate and assemble. This clearly suggests that the GUV membrane is unilamellar and functional for the integration of membrane proteins. Furthermore, the fluorescence inside the GUVs remained the same in the absence of alpha‐hemolysin, an observation that also confirms the stability (i.e., no bursting or leakage) of the GUVs.

**Figure 8:**
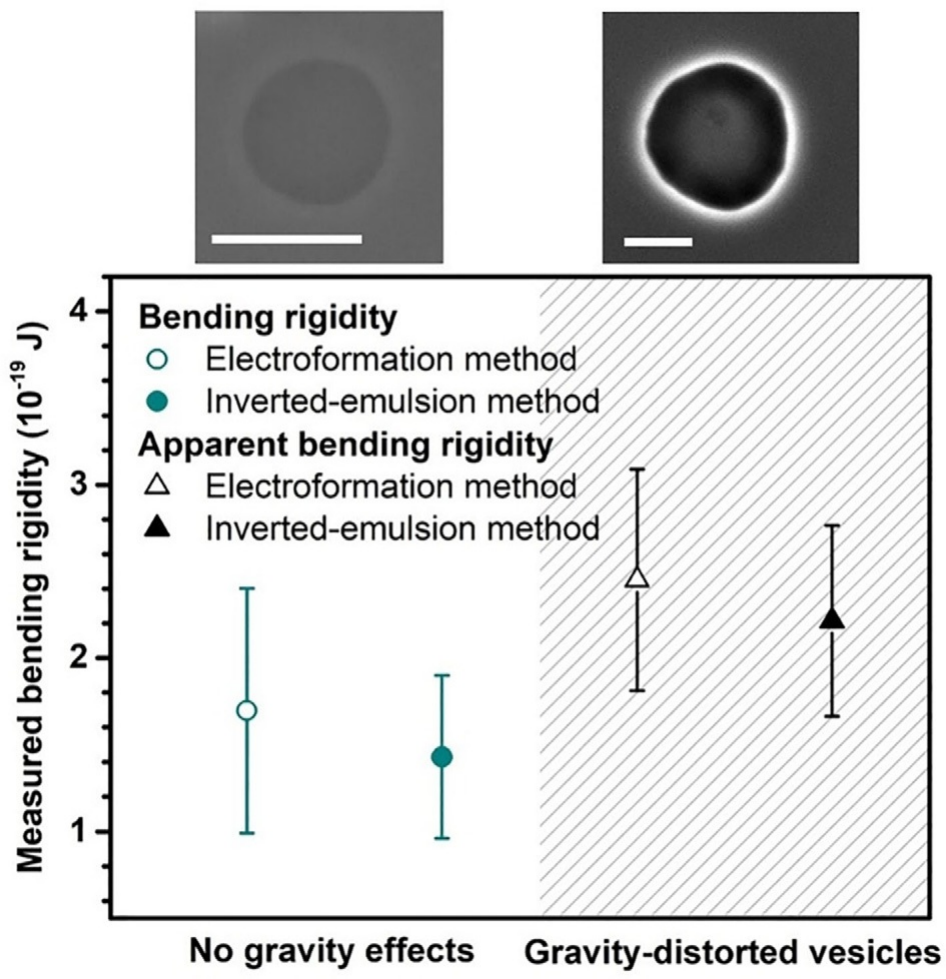
Bending rigidity measurements of POPC GUVs produced by electroformation (open symbols) and inverted emulsion methods (solid symbols). The vesicle interior contains 600 mOsm sucrose. Data are shown for preparations where the gravity effects were overcome by replacing the external 600 mOsm glucose solution (black symbols; hatched area) with a solution of 25 mOsm glucose and 575 mOsm sucrose (dark cyan). Mean data are shown with error bars taken from the standard deviation (*n*≥10). Snapshots above the graph show phase contrast images of GUVs under the respective conditions. Note that for 600 mOsm glucose in the outer solution, the contrast is enhanced due to an increased refractive index difference. Scale bars: 50 μm.

An ancillary measurement to understand the composition and purity of lipid membranes is to measure their bending rigidity.^50^ Here, we employed the method of fluctuation spectroscopy as described in Gracia et al.^51^ However, for systems with high sugar content as used here, one should be aware of two issues: 1) sugars decrease the bending rigidity of GUV membranes^50^, ^52^, ^53^ and direct comparison with literature data should take this into account; and 2) at high sucrose/glucose contrasts, the density difference across the membrane distorts the vesicle shape and can affect the membrane fluctuation spectrum leading to errors in the assessed bending rigidity.^54^ Vesicles containing 600 mOsm sucrose in their interior and suspended in equimolar glucose solutions are affected by both of these effects. To avoid the error associated with gravity, which often appears to be neglected when comparing inverted emulsion with electroformed GUVs (see, e.g., ref. 38), we decreased the sucrose/glucose density gradient. For this, the external GUV solution was adjusted to 575 mm sucrose and 25 mm glucose. This condition ensures no gravity‐associated error as assessed from the gravitational parameter introduced in Henriken and Ipsen.^54^ The data presented in Figure 8 show that there is no significant difference between the bending rigidities of the GUVs produced using electroformation (1.70±0.71)×10^−19^ J and the inverted emulsion (1.42±0.47)×10^−19^ J methods. For comparison, we also plot the data obtained without the gravity correction showing an erroneous apparent increase in the bending rigidity. The bending rigidity of inverted‐emulsion‐based vesicles appears slightly lower than that of electroformed vesicles but the difference is not significant (confirmed with a t‐test). These experiments ascertain that the lipid membranes produced by the inverted emulsion method are comparable to those of GUVs produced from conventional techniques.

## Conclusion

The inverted emulsion‐based method is relatively a new technique for producing giant vesicles when compared to electroformation or gentle hydration. This particular method of producing giant vesicles, leaflet‐by‐leaflet, has series of advantages. Although the major benefit is the possibility to encapsulate large (bio)molecules inside the GUVs under physiological conditions, the method can be optimized to produce GUVs with minimum preparation times and for high‐throughput analyses. In this work, we have optimized various parameters to reduce the time required to produce the GUVs. This was made easy due to the use of 96‐well microtiter plates for parallel preparations/experiments. Our results demonstrate that density gradients by using sugars are vitally important in manipulating the overall yield of the GUVs. Alternatively, polymers, such as PEG or PVA, can be used to replace inner sugar solutions, if necessary. We have also explored the possibility of using different oils for solubilizing the lipids because having a homogenous lipid solution in the carrier oil free from aggregation is important for the formation of the interfacial lipid monolayer. From our findings it is evident that mineral oil is the most suitable amongst all the other oils (see Table S1 for more details). Another significant finding of this work is that, with the exception of pH 12, there is little effect of pH and temperature on the yield and size of the GUVs at least for the lipid composition tried here. This is beneficial for encapsulating components that are active only at specific pH and temperature conditions. For example, it is possible to encapsulate enzymes inside the GUVs at low temperatures when they are inactive and activate them later under the microscope to study the kinetics of conversion and the effect of the resultant molecules on the lipid bilayer. We also note the robustness of using the 96‐well plate to perform the inverted emulsion procedure. This is evidenced by the relatively small error bars for each experiment and when 600 mOsm sucrose is used the GUV yields and sizes are comparable across experiments.

When implementing the inverted emulsion method in microtiter plates, the following conditions can be used for optimal GUV yields: 20 μL of mineral oil with 200 μm lipids to form the interface over 600 mOsm outer glucose solution for a period of 30 min. This is followed by 7 μL inner sucrose solution at 600 mOsm mixed with 250 μL lipid mineral oil to form the emulsion. Then 50 μL emulsion is added on top of the interface and subjected to centrifugation at 200 *g* for a period of 3 min. The total time taken for optimal production of GUVs is 35 min. We also calculated the minimum time needed to produce GUVs, which was 18 min by reducing the incubation period from 30 to 15 min. Note that in this case a suboptimal number of vesicles is obtained but it is better suited for time‐sensitive experiments.

The most likely hurdle for widespread acceptance of this technique could be the possibility of oil present in the lipid bilayer that could potentially alter the biophysical properties of the membrane. To this end, we have addressed this concern (at least for our setup) by incorporating a functionally active membrane protein. Alpha‐hemolysin in its monomeric form in solution can assemble as a heptamer into the lipid bilayer to form a pore.^55^, ^56^ Not only have we showed that this is possible within the lipid bilayer of the GUVs produced by using an inverted emulsion, but also that the time and concentration required for total loss of fluorescence inside the GUVs is comparable to that of electroformed GUVs.^26^ Along with this assay, our membrane bending rigidity analysis of these GUVs supports the conclusion that the GUVs are oil‐free (or, at least, that mineral oil does not alter their mechanical properties) and are therefore biomimetic. Furthermore, performing the method in 96‐well plates provides the advantage of scalability for greater statistically significant results and high‐throughput experiments. Overall, we believe that our findings will aid in the widespread adaptation of this method for membrane studies where high encapsulation efficiencies, physiological conditions and biomimetic GUVs are required.

## Experimental Section

**Materials**: 1‐Palmitoyl‐2‐oleoyl‐*sn*‐glycero‐3‐phosphocholine (POPC) was purchased from Avanti Lipids Polar, Inc., Alabaster, AL. 1,1′‐Dioctadecyl‐3,3,3′,3′‐tetramethylindocarbocyanine perchlorate (DilC18) to label the membranes from Invitrogen, calcein as a fluorescent dye to stain the interiors of GUVs from Thermo Fisher Scientific Inc. Polyvinyl alcohol (PVA), fully hydrolyzed, with a molecular weight of approximately 145 kDa was purchased from Merck (Germany) and polyethylene glycol (PEG), with a molecular weight of approximately 8 kDa crystalline, from Sigma–Aldrich (Germany). 96‐Well microtiter plates, optically clear flat bottom, were from Corning. Alpha‐hemolysin, bovine serum albumin (BSA) and β‐casein (from bovine milk) were obtained from Sigma–Aldrich (Germany). Other chemicals including organic solvents (chloroform, acetone, and ethanol), sugars (glucose and sucrose) and buffers (PBS, Tris, and HEPES) were purchased from Sigma–Aldrich (Germany).

**Lipids in oil preparation**: Chloroform‐solubilized POPC (99.9 %) and DilC18 (0.01 %) were used to make lipid–oil solution. Briefly, the preparation of lipid–mineral oil solution starts with forming a thin film of dried lipids in a clean glass vial by evaporating the chloroform under argon flow. A desiccator was used to dry the lipids for a period of 1 h and remove any leftover traces of chloroform. Finally, mineral oil was added to the glass vial under low humidity (<10 %) conditions using an AtmosBag (Sigma–Aldrich) filled with nitrogen gas and monitored with a humidity gauge (Klimalogg Pro, TFA Dostmann). This was followed by a sonication step for better solubilization of lipids in the oil. At this stage, the total concentration of the lipids in the mineral oil can be from 50 to 600 μm, depending on the amount of mineral oil added. The lipid–mineral oil solution was then incubated overnight at room temperature in the dark to ensure that the lipids are completely dissolved. These lipid–oil solutions can be stored for up to one week at 4 °C and brought to room temperature before usage.

**Surface treatment of the microtiter well plates**: To achieve high‐throughput and parallelized experiments, the entire inverted emulsion method has been performed in 96‐well microtiter plate. Surface treatment of the glass bottom of the wells is important to avoid adhesion and bursting of the GUVs. Wells within the plates are pre‐coated using aqueous solutions of β‐casein (2 mg mL^−1^). Typically, 30 μL of the coating solution was added to each well and allowed to dry in the presence of vacuum for 30 min. The well was gently washed multiple times with the outer solution before making the lipid interface. BSA at 2 mg mL^−1^ was used as a coating material in experiments involving high (12) or low (4) pH where the β‐casein coating is not stable.^57^

**Inner and outer aqueous solutions**: All aqueous solutions were made using MilliQ Millipore® water. Inner solutions contained either sucrose (50, 300, 600, and 900 mm) or PEG or PVA at 0.5, 1, 2.5, 5 *w*/*v* %. For pH‐based experiments, the solutions were buffered by using HEPES at 20 mm concentration and NaOH as well as acetic acid were used to adjust the pH. Inner solutions were also made with either HEPES (50, 100, 200, 400 mm), Tris (50, 100, 200, 400 mm), or PBS, and sucrose was added to provide enough density. For all the above inner solutions, corresponding concentrations of glucose solutions were used as the outer solutions to maintain isoosmotic conditions across the membrane.

**Inverted emulsion method**: Initially, 50 μL of glucose (outer) solution were added to the pre‐coated wells in the microtiter plate. This was followed by the addition of 20 μL of the lipid–oil mixture on top of the glucose. The entire setup was allowed to incubate for a period of 5, 15, 30, or 60 min to form an interfacial lipid monolayer (surface area of ∼192 mm^2^). Then 250 μL of the same lipid–oil mixture were added to a 1.5 mL Eppendorf® tube. To this tube, 1, 3, 5, 7, 10, or 25 μL inner solution were added and then agitated mechanically along a standard Eppendorf tube rack to yield a water‐in‐oil emulsion. Note that four emulsion preparation techniques were compared. Figure S8 shows that mechanical agitation yields the narrowest size distribution compared to vortexing and pipetting by hand. Sonication did not provide any useable droplets. We also show that the sizes of the emulsions result in similar sizes of the final GUVs (Figure S8 b). Based on these findings and the fact that sonication and vortexing can cause protein degradation, we used mechanical agitation in this work, but for some applications vortexing may be sufficient. An aliquot of 50 μL of the emulsion was pipetted into the wells containing the lipid monolayer interface. Immediately after this, the microtiter plate was transferred into a centrifuge. Based on the experiment either 50, 100, 200, 300, 400, 600, or 1200 *g* force was applied for periods of 30 s, 1 min, 3 min, or 10 min.

**Membrane composition and functionality**: GUVs containing 10 μm of the fluorescent dye calcein were incubated with 2.5 μg mL^−1^ alpha‐hemolysin for a period of 30 min. Confocal fluorescence images were acquired before and after the assay. For bending rigidity measurements, GUVs containing 600 mOsm sucrose inside and 600 mOsm glucose outside were prepared by using the inverted emulsion method and then diluted with sucrose to achieve an out solution of 575 mOsm sucrose and 25 mOsm glucose as required. Electroformed GUVs were produced by using a procedure explained elsewhere with minor modifications: 2 V_p‐p_ at 10 Hz for a period of 3 h in 600 mOsm sucrose and diluted as above when required.^55^ Measurements and subsequent analysis for bending rigidity measurements have been performed by using the methods developed by Garcia et al.^51^ Acquisitions were performed by using an inverted microscope (Axiovert 135 Zeiss, Germany) equipped with 20× objective and a fast digital camera (eCMOS PCO.edge, PCO AG, Germany) with an exposure time of 200 μs at 20 frames per second.

**Microscopy**: The produced GUVs can be directly observed within the microtiter plate using an inverted microscope without any further sample preparation. Here, the samples were observed and images acquired using a confocal microscope (Leica microsystems TCS SP8, Wetzlar, DE) equipped with a 63×/1.4 NA water immersion objective. A total of six confocal images were randomly taken in each well and with the same field of view for comparable yield assessments. The diameter and population of individual GUVs were measured using the Vesicle‐Analyser‐Pro software.^58^ Note that multiple *z*‐stacks were acquired at each position to ensure all vesicles were imaged and that each vesicle was measured at the equatorial plane. GUVs with diameters below 10 μm were excluded. DiIC_18_ fluorescence in the membrane was excited by using a 552 nm diode laser with emission collected at 562–635 nm and calcein present inside the GUVs by a 488 nm diode laser with emission collected at 498–535 nm.

## Conflict of interest

*The authors declare no conflict of interest*.

## Supporting information

As a service to our authors and readers, this journal provides supporting information supplied by the authors. Such materials are peer reviewed and may be re‐organized for online delivery, but are not copy‐edited or typeset. Technical support issues arising from supporting information (other than missing files) should be addressed to the authors.

Supplementary
