# Supplementary material for: Optimization of the Inverted Emulsion Method for High‐Yield Production of Biomimetic Giant Unilamellar Vesicles
Source: Chembiochem. 2019 Oct 11;20(20):2674–82. doi: 10.1002/cbic.201900529 (PMC6856842; doi:10.1002/cbic.201900529)
Supplement: Supplementary file 1 — Supplementary [file CBIC-20-2674-s001.pdf]

CHEMBIOCHEM

Supporting Information

Optimization of the Inverted Emulsion Method for High-Yield Production of Biomimetic Giant Unilamellar Vesicles

Akanksha Moga, Naresh Yandrapalli, Rumiana Dimova, and Tom Robinson*[a]

cbic_201900529_sm_miscellaneous_information.pdf

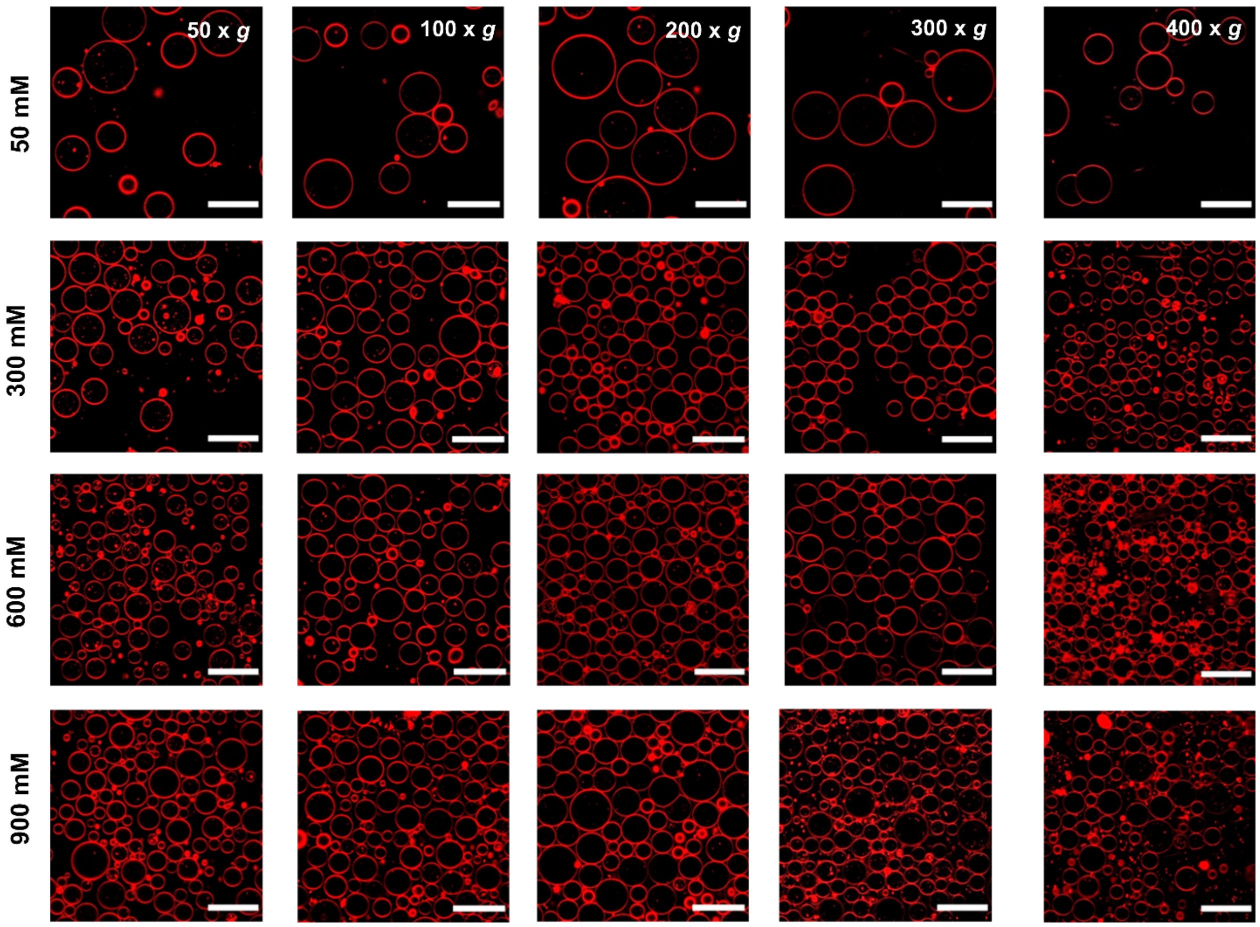

Figure S1. Confocal images of GUVs containing various concentrations of sucrose solutions produced using different centrifugal forces. The results are obtained with for osmotically matched glucose as the outer solution and with centrifugation speeds applied for 3 minutes at RT. The incubation time for the interface formation was 30 minutes with a lipid concentration of 400 µM (n=3). Scale bar: 50 µm

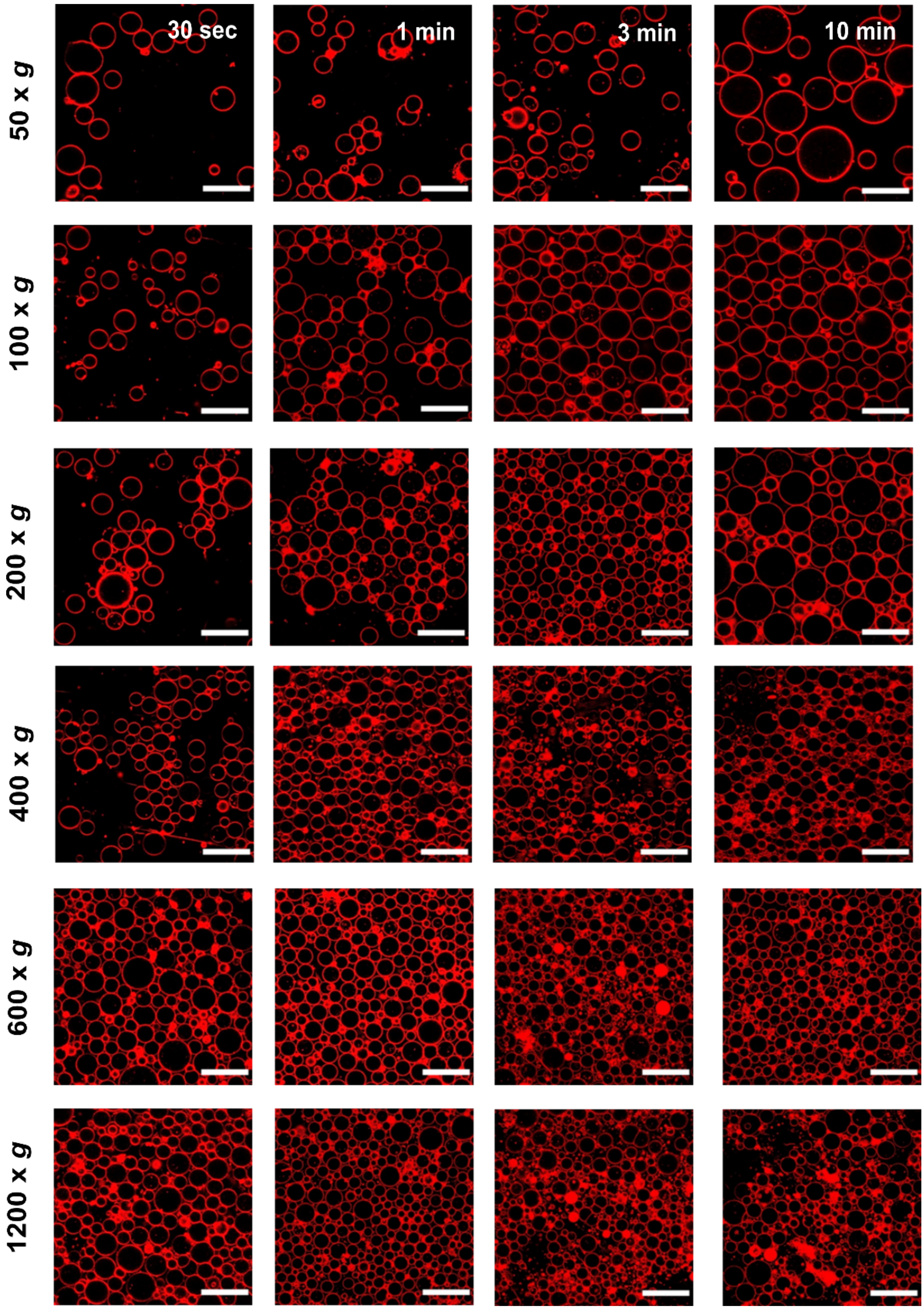

Figure S2. Confocal images of GUVs containing 600 mM concentration of sucrose solution produced with different centrifugal forces for various time periods. Osmotically matched glucose was used as the outer solution. Results were obtained for a fixed 400 µM lipid concentration and 30 minutes interfacial incubation time at RT (n=3). Scale bar: 50 µm.

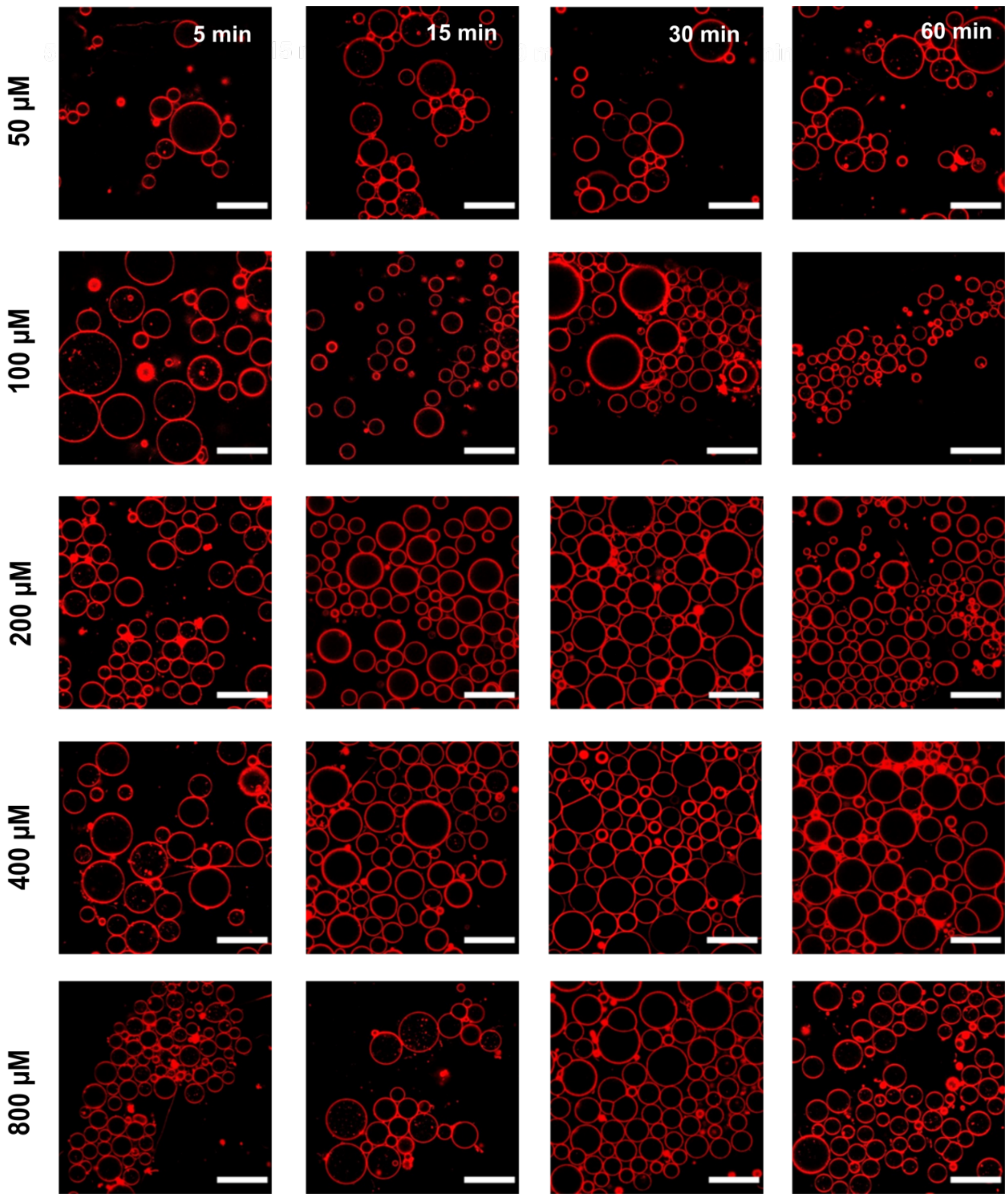

Figure S3. Confocal images of GUVs produced with various incubation periods for the monolayer formation and for different concentrations of lipids in mineral oil. Results are obtained for a fixed 600 mM sugar solution and at 200 x g centrifugation speed for 3 minutes at RT (n=3). Scale bar: 50 µm.

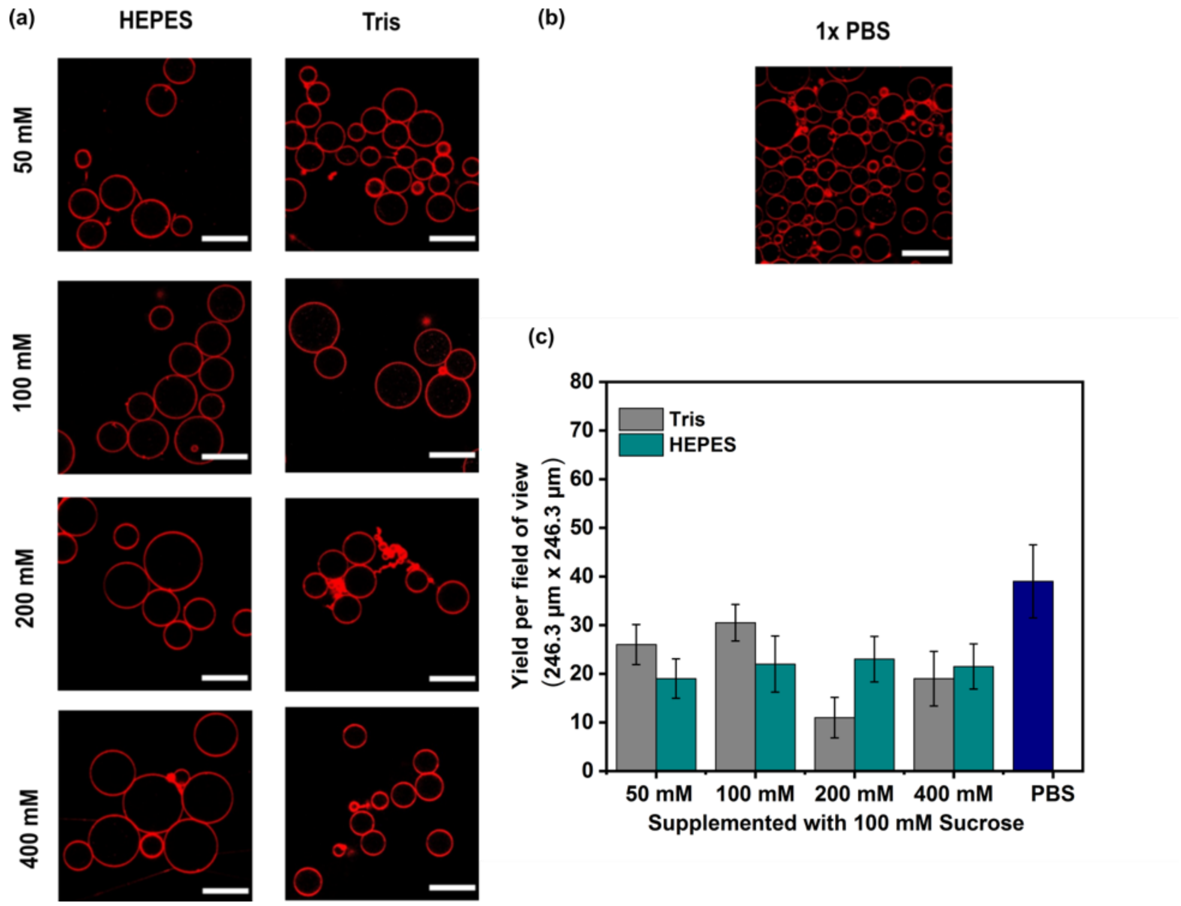

Figure S4. Confocal images of exemplary GUVs produced with varied concentrations of (a) HEPES and Tris buffers or (b) 1 × PBS as the inner solution only. (c) The yields of GUVs produced for various concentrations of HEPES or Tris as well as with 1 × PBS. Note that 100 mM sucrose was added to the inner solution to provide a density gradient and individually osmotically matched glucose was used as the outer solution. Results were obtained for fixed 100 mM sugar solutions, 200 μM lipid concentration and 30 minutes interfacial incubation at 200 x g centrifugation speed with 3 minutes at RT (n=3). Scale bar: 50 μm.

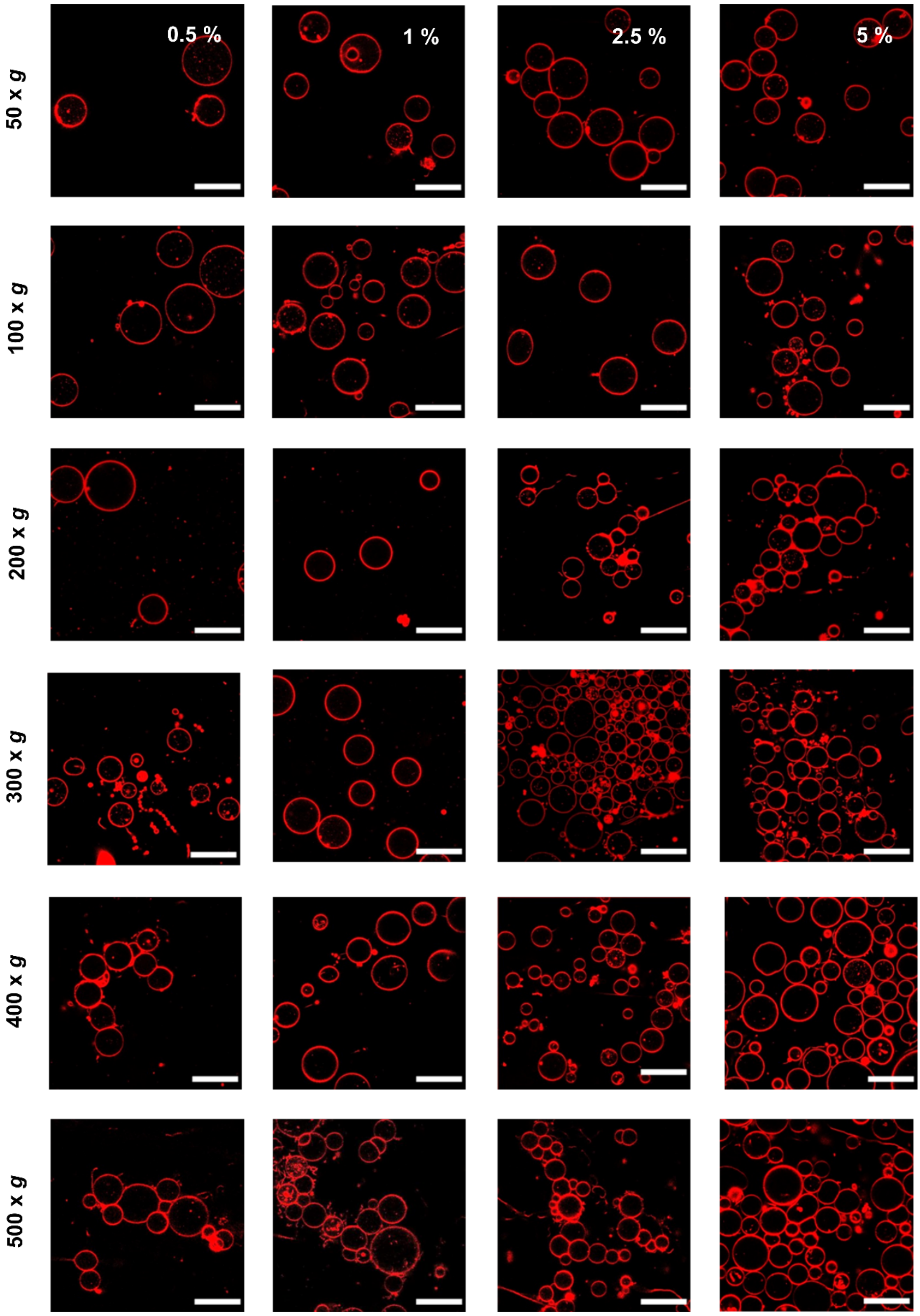

Figure S5. Confocal images of GUVs produced with various concentrations of PVA as the inner solutions at different centrifugation speeds. Results were obtained for a fixed 200 µM lipid concentration and 30 minutes interfacial incubation time at RT (n=3). Scale bar: 50 µm.

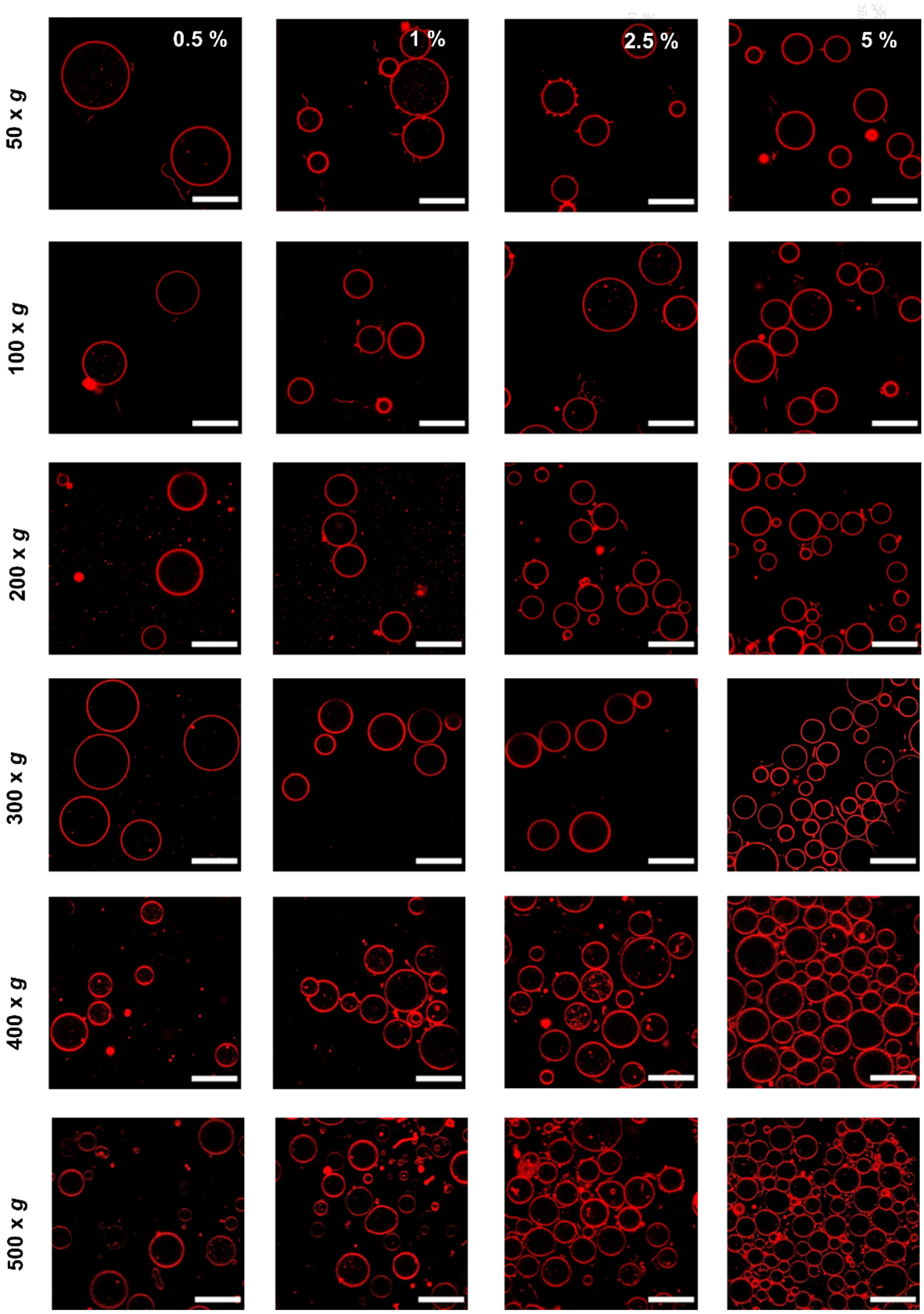

Figure S6. Confocal images of GUVs produced with various concentrations of PEG as the inner solutions at different centrifugation speeds. Results were obtained for fixed 200 µM lipid concentration and 30 minutes interfacial incubation time at RT (n=3). Scale bar: 50 µm.

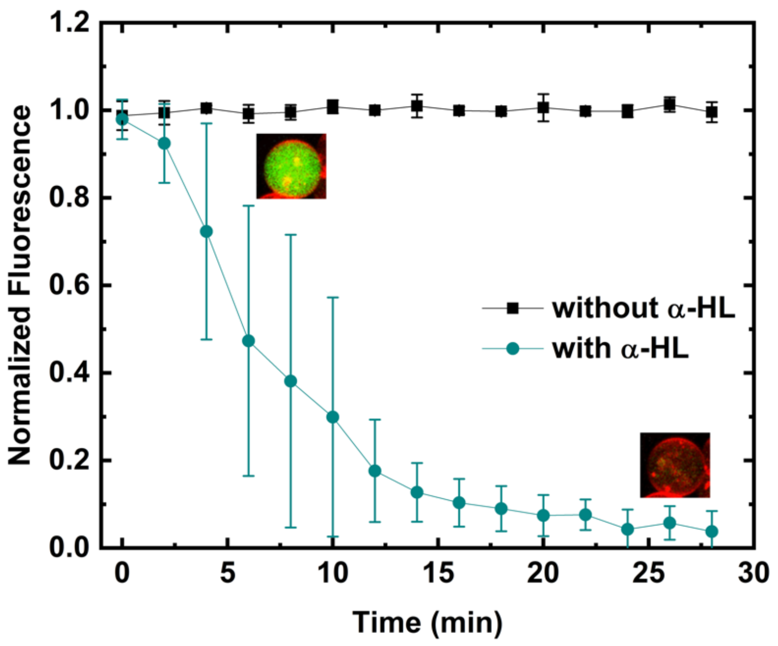

Figure S7. Membrane permeation assay using 2.5 µg/ml concentration of alpha-hemolysin incubated with GUVs encapsulating 10 µM calcein produced using the inverted emulsion method. The mean internal intensity is given with error bars taken from the standard deviations (n=10). Inset showing an exemplary GUV before and after addition of alpha-hemolysin and pore formation. GUVs were captured and solutions exchanged in < 1 min using a microfluidic device detailed elsewhere [1]. GUVs were prepared using the following conditions: 400 µM lipid concentration, 30 minutes interfacial incubation time, inner solution volume of 5 µl in 250 µl lipid-oil, 200 *x g* for 3 min, and 600 mOsm sugars at RT.

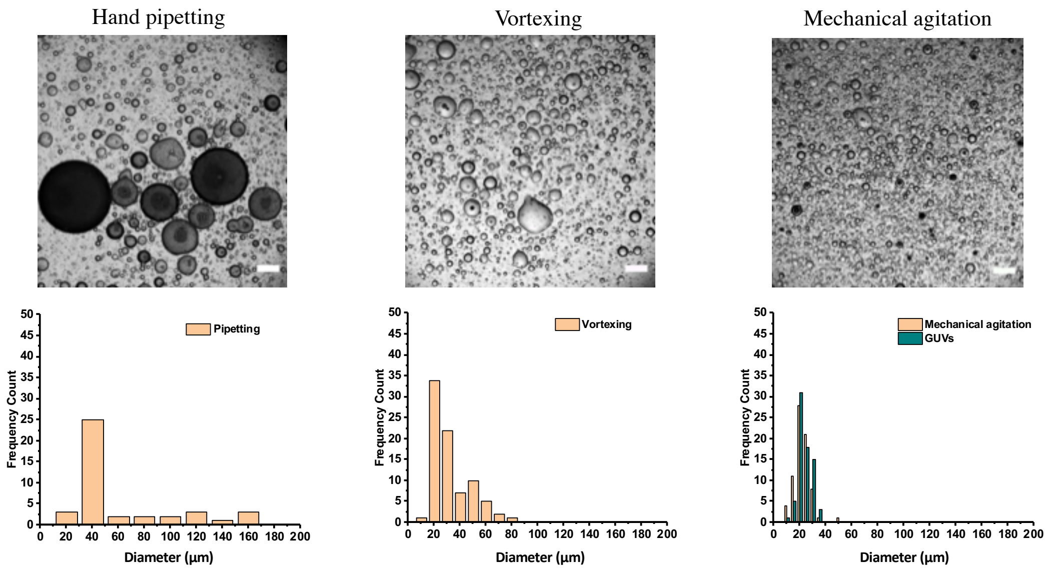

Figure S8. Comparison of emulsion preparation techniques. a) Bright-field images of emulsions on a glass coverslip. Compared to mechanical agitation, vortexing and hand pipetting yielded ~ 60% and ~ 44% droplets respectively. Scale bar: 50 µm. b) Histograms of the size distributions of the emulsions. Final vesicle sizes for the mechanical agitation are also including to verify that the sizes are conserved from droplets to GUVs.

Osmolarity of sugar solution (mOsm)	Speed \ Oil	1-Octanol	Oleic acid	Anisole	Silicon oil	Squalene	Mineral oil
10	50 × g	No GUVs	No GUVs	No GUVs	No GUVs	No GUVs	No GUVs
100	50 × g	No GUVs	No GUVs	No GUVs	No GUVs	No GUVs	No GUVs
400	50 × g	No GUVs	No GUVs	No GUVs	No GUVs	No GUVs	Few GUVs
900	50 × g	No GUVs	No GUVs	No GUVs	No GUVs	No GUVs	Few GUVs
10	100 × g	No GUVs	No GUVs	No GUVs	No GUVs	No GUVs	No GUVs
100	100 × g	No GUVs	No GUVs	No GUVs	No GUVs	No GUVs	Few GUVs
400	100 × g	No GUVs	No GUVs	No GUVs	No GUVs	No GUVs	Few GUVs
900	100 × g	No GUVs	No GUVs	No GUVs	No GUVs	No GUVs	Good number and size
10	200 × g	No GUVs	No GUVs	No GUVs	No GUVs	No GUVs	No GUVs
100	200 × g	No GUVs	No GUVs	No GUVs	No GUVs	No GUVs	Few GUVs
400	200 × g	No GUVs	No GUVs	Few GUVs	No GUVs	No GUVs	Good number and size
900	200 × g	No GUVs	No GUVs	Few GUVs	No GUVs	No GUVs	Good number and size
10	300 × g	No GUVs	No GUVs	Few GUVs	No GUVs	No GUVs	Good number and size
100	300 × g	No GUVs	No GUVs	Few GUVs	No GUVs	No GUVs	No GUVs
400	300 × g	No GUVs	No GUVs	Few GUVs	No GUVs	No GUVs	Good number and size
900	300 × g	No GUVs	No GUVs	Few GUVs	Few GUVs	No GUVs	Good number and size
10	400 × g	No GUVs	No GUVs	Few GUVs	No GUVs	No GUVs	No GUVs
100	400 × g	No GUVs	No GUVs	Few GUVs	No GUVs	No GUVs	Good number and size
400	400 × g	No GUVs	No GUVs	Few GUVs	Few GUVs	Few GUVs	Good number and size
900	400 × g	No GUVs	No GUVs	Few GUVs	Few GUVs	Few GUVs	Few GUVs

Legend: No GUVs · Few GUVs · Good number and size

Table S1: Showing that the choice of oil for solubilizing the lipids is a crucial step in the production of inverted emulsion-based method. In this work, mineral oil provided the best results compared to any other oil tested. Results were obtained for a fixed 200 µM lipid concentration, 30 minutes interfacial incubation time, and 3 min centrifugation time at RT (n=3).

[1] N. Yandrapalli, T. Robinson, *Lab Chip* **2019**, DOI 10.1039/c8lc01275j.
